# Targeting the TGFβ pathway with galunisertib, a TGFβRI SMI, promotes anti-tumor immunity leading to durable, complete responses, as monotherapy and in combination with checkpoint inhibition

**DOI:** 10.1186/2051-1426-3-S2-P402

**Published:** 2015-11-04

**Authors:** David Schaer, Yanxia Li, Stephen Castaneda, Ivan Inigo, David Surguladze, Xiaohong Xu, Desiree Nugent, Mary Murphy, Gerald Hall, Karim Benhadji, Susan Guba, Yiwen Li, Michael Kalos, Kyla Driscoll

**Affiliations:** 1 Eli Lilly & Company, New York, NY, USA; 2 Eli Lilly & Company, Indianapolis, IN, USA

## 

TGFβ signaling is known to play a central role in tumor biology, via inducing and/or enhancing tumor cell growth and differentiation, modulating the extracellular matrix in the stroma, inducing epithelial to mesenchymal transition, modulating angiogenesis, and inhibiting immune surveillance and anti-tumor immunity. Galunisertib is a pharmacological inhibitor of the TGFβ pathway which acts by inhibiting signaling though TGFβRI. Galunisertib is currently being evaluated clinically in several Phase I and II studies; as a monotherapy, galunisertib has shown antitumor activity against a variety of tumors, including durable and long-term responses in patients with glioma.

To explore the impact of Galunisertib monotherapy on anti-tumor T cell immunity, we utilized murine tumor models. Treatment of mice with well-established 4T1-LP (poorly immunogenic 4T1 breast tumor engineered to express luciferase) implanted in the mammary fat pad resulted in strong dose-dependent anti-tumor activity with nearly 100% inhibition of tumor growth across doses during the dosing period, with complete tumor responses upon cessation of treatment in ~50% of animals at the highest dose tested; depletion studies demonstrated that regression of 4T1-LP was dependent on the presence of CD8+ T cells. Rechallenge of treated, tumor free mice resulted in complete rejection of 4T1-LP tumor cells but no rejection of EMT6-LM2 tumor cells, demonstrating the establishment of a durable response and immunological memory. Treatment of mice bearing established parental 4T1 tumors in the mammary fat pad resulted in no significant inhibition of tumor growth, indicating that the presence of a foreign antigen (i.e. LP), potentially enhanced the ability to regress the 4T1-LP derivative. Animals that rejected the immunogenic 4T1-LP tumors were able to also reject 4T1 parental cells upon rechallenge, suggesting the development of a secondary immune response via antigen spreading as a consequence of effective tumor targeting. In the CT26 murine colon carcinoma model, treatment of established tumors with galunisertib or anti-PD-L1 as monotherapies resulted in tumor growth inhibition compared to control of 75% and 86%, respectively (T/C values of 25% and 14%); complete responders were observed in about 20% of treated animals in both monotherapy groups. Combination of galunisertib with anti-PD-L1 resulted in an enhanced tumor growth inhibition of 98% (T/C value of ~2%), and a complete response rate of ~50%, suggesting at least additive activity with potential for synergy when targeting the TGFβ and PD-1 pathways. Taken together, these data demonstrate the potential for galunisertib treatment to enhance the development of anti-tumor T cell immunity, which can be enhanced by combinations with immune check point inhibitors.

